# Nursing shared governance at hospitals – it’s Finnish future?

**DOI:** 10.1108/LHS-10-2018-0051

**Published:** 2019-09-26

**Authors:** Taina Hannele Kanninen, Arja Häggman-Laitila, Tarja Tervo-Heikkinen, Tarja Kvist

**Affiliations:** 1 Department of Nursing Science, University of Eastern Finland, Kuopio, Finland; 2 Clinical Nurse Consultant, Kuopio University Hospital, Kys, Finland; 3 Department of Nursing Science, University of Eastern Finland, Kuopio, Finland

**Keywords:** Nursing, Hospital, Shared governance, Nurse, Council, Meeting document

## Abstract

**Purpose:**

The purpose of this study is to describe council structure, its benefits, supportive and obstructive factors and developmental needs as a part of shared governance in a university hospital.

**Design/methodology/approach:**

This is a descriptive study, where semi-structured interviews with 12 nurses was conducted in 2014 and documents from 75 council meetings from 2009 to 2014 were gathered and analyzed. Qualitative content analysis method was used on the data.

**Findings:**

The study hospital has been developing nursing shared governance with unique structure and processes of councils. Professors and university researchers act as chair and members are voluntary nursing staff. The factors supporting the councils are nurse managers’ support, enthusiastic personnel and neighboring university. The factors obstructing the councils are lack of time, understanding and skills. The work of the councils benefits the organization by improving patient care, harmonizing nursing practices and informing decision-making. The council’s developmental needs were more visibility, concentration into everyday problems and interprofessionality.

**Research limitations/implications:**

Applying nursing shared governance structures into an organization improves the professional practice environment of nursing personnel.

**Practical implications:**

The study hospital has its own, unique council structure. It did not cover the whole hospital or all of the nursing personnel, but it is already producing promising results. It should be given an official status and more support from nurse managers, and it should be developed into an inter-professional discussion. The results presented here indicate that shared governance, even, in its early stage, contributes positively to the quality of care, harmonizes nursing practices and informs decision-making. Applying shared governance structures into an organization improves the professional practice environment of nursing personnel. The study showed concrete supporting and obstructing factors that should be notified in nursing leadership.

**Originality/value:**

Despite the extensive empirical studies on nursing shared governance, there is very little research on councils in the Scandinavian countries.

## Introduction

Shared governance is a leadership strategy where staff nurses and nurse leaders work together to develop nursing practice. The principle idea of shared governance is, that use of power and decision-making should be done in the right place (Porter-O’Grady, 1997). The decision-making is delegated to provide an opportunity for staff nurses to exercise control over their practice (Fray, 2011; Hess, 2017).

Porter-O’Grady *et al.* (1997) says, that the sustainability of health care depends on how well or poorly, nurses work in the point-of-service. When nurses exercise control over decisions, their work is integrated with management decisions to achieve desired and mutually agreed outcomes (Clavelle *et al.*, 2013). Nurses are seen able and competent to work independently, and their professionalism and readiness to accept accountability is highly recognized in the organization. Nurse leaders’ role is to give support, arrange recourses and maintain limitations. Staff and leaders create strategic plans, shared governance by-laws, and budgets together (Swihart, 2011).

Shared governance was first introduced in nursing during the 1980s, when the first tentative models where constructed. These models include a committee/council structure to provide venues for nurses to partner with other colleagues in addressing clinical and administrative challenges (Rheingans, 2012). After that, shared governance has spread with Magnet Recognition program®. Hospitals that are seeking Magnet status are required to demonstrate a system of shared decision-making and participatory management (Porter-O’Grady and Finnigan, 1984; American Nurses Credentialing Center, 2008; Kvist *et al.*, 2013).

The realization of shared governance models has adapted to different organizations in different environments (Hess, 2004; Porter-O’Grady, 2019). Each organization has its own culture and structure that effect the creations of suitable models (Porter-O’Grady, 1991). The best way to find a suitable model is a joint effort between the administration/managers and staff (Lindell Joseph and Bogue, 2018).

There has been a lot of research into the shared governance structures. Profit Newman (2011), Burkman *et al.* (2012) and Cadmus *et al.* (2015) explain how to select and to construct a suitable structure. Others describe the structures they have selected (Meyers and Costanzo, 2015; Powers and Thornton Bacon, 2016) or modified (Whitt *et al.*, 2011; Gallagher-Ford, 2015). The chosen model of shared governance can become stalled over time and needs organizational support to move forward (Gerard *et al.*, 2016). It can be strengthened with different kinds of new structures like unit councils (Duncan and Hunt, 2011; Jordan, 2016) or journal clubs (Palaka *et al.*, 2011; Häggman-Laitila *et al.*, 2016) or by using LEAN-methodology to increase cross-council communication (Moreno *et al.*, 2018). Shared governance can be applied to night time nursing (Winks *et al.*, 2017) or taken outside hospitals to ambulatory care (Powers and Thornton Bacon, 2016). There have been reports of the functioning and the results of the councils (Fray, 2011) and the positive effects it has had in the job satisfaction and careers of the nurses (Brody *et al.*, 2012).

It has been shown that shared governance improve work environments and empowerment of the nurses (Clavelle *et al.*, 2013; Di Fiore *et al.*, 2018,) and increase nurses retention (Fisher *et al.*, 2016) by strengthening collaboration and shared decision-making both inside the unit and between disciplines (Kramer *et al.*, 2011). Empowerment supports the commitment of the personnel by giving them a chance to use their professional abilities (Li *et al.*, 2013; Yang *et al.*, 2013). When nurses can express their ideas and thoughts to the leaders, leaders can get vital information to support decision making and nurses, on the other hand, can see the results of their own ideas (Whitt *et al.*, 2011). Studies have also demonstrated that control over their own work will enable nurses to develop their skills (Brody *et al.*, 2012; Rheingans, 2012). This leads to better quality of care and safety of the patients (Cadmus *et al.*, 2015) which then again leads to cost saving (Whitt *et al.*, 2011; Brody *et al.*, 2012; Rheingans, 2012).

In Finland, shared governance is still relatively new (Mäntynen *et al.*, 2014). The study hospital was chosen for this study because it has used the Magnet ideology (Mäntynen *et al.*, 2014) to develop its work since 2006 and introduced the first nursing councils in co-operation with the neighboring university in 2008. Even though council structures do not yet cover the whole hospital, they have been in operation long enough to be evaluated.

**Figure 1.**
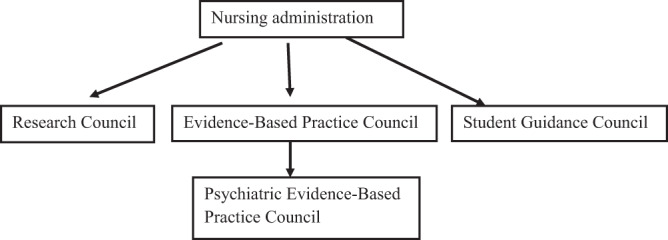
The council structures of the study hospital

In 2014, the Finnish study hospital had a Research Council, an Evidence-Based Practice Council, a Student Guidance Council, and a Psychiatric Evidence-Based Practice Council. The Research Council started in year 2008 and its purpose is to promote scientific research in health sciences and the use of evidence-based-nursing in the area. The Evidence-Based Practice Council started in 2010 and its purpose is to promote evidence-based-nursing and the expertise of nurses. The Psychiatric Evidence-Based Practice Council was founded in 2009 after the first course in evidence-based-nursing was initiated in the organization. Its purpose is to promote evidence-based-nursing in psychiatry and concentrate on developing psychiatric nursing. The Student Guidance Council was founded in 2011 and it strives to serve as a venue for all parties in student guidance to come together (Figure 1).

A qualitative approach was chosen as method for this study, because, even though internationally shared governance has been studied largely, in Nordic Countries, it is still not commonly used and the existing nursing councils in Finland have been created as a result of various organizational development programs. Their functioning has not yet been studied and their impact in nursing is not known. This study concentrates in the views of the nurses participating in the councils and to the written documents of the council meetings.

## Aim

The aim of this study is to describe council structures, its benefits, supportive and obstructive factors and developmental needs as a part of shared governance in one university hospital.

The research questions were:

RQ1. What kind of council structures and processes can be found?

RQ2. What factors do the members of councils regard as supportive or obstructive for the functioning of the councils?

RQ3. How have the councils as part of shared governance structures benefitted the study hospital?

RQ4. What are the developmental needs of councils?

## Methodology

### Study design

This study is a qualitative descriptive interview and document study. As the organized information about the councils in the study hospital was scarce, analyzing the documents revealed the routines of the councils. The decision to use interviews as data source along with documents was a natural choice, as we wanted to hear each participant’s unique experience about the work of the councils.

### Participants and research context

The study site is one of five university hospitals in Finland. It provides the best possible care to patients in all fields of specialized medical care and engages in internationally recognized research. The hospital is one of the largest trainer of physicians in Finland, and one of the largest health-care industry teaching hospitals in the country with 4,300 employees and 90,000 treated patients per year.

The participants were recruited based on the criteria that they had participated in the work of the councils. The first to be interviewed were the chairs of the councils and they suggested members for the following interviews.

The participants were all nurses, spoke Finnish and were working in either the hospital or university. Some of them had advanced degrees from universities. They worked as nurse leaders, professors, researchers, clinical experts, or staff nurses. In shared governance structures, they worked as chair, secretary or member. Participants were fully informed of the study and were required to give their written consent form before participating.

### Data collection

Semi-structured interviews of 12 nurses were performed between April and August in 2014. The themes of the interviews were the functioning of the existing councils, factors supporting or obstructing the councils and benefits and developmental needs of shared governance. The interviews ranged in length from 20 to 40 minutes. Interviews were digitally recorded, professionally transcribed (73 pages), and checked by the principal researcher. Most interviews were held in private offices.

Along with the interviews, the chairs of the councils were also asked for documents such as memos and minutes from the meetings. Altogether, there were 75 meetings and 150 pages of memos. In total, 171 people took part in the work of the councils during years 2009-2014. The total amount of data was 223 pages.

### Data analysis

The documents were analyzed by gathering information about the work of the councils, for example who acted as chair, and how often the meetings where held and what issues were dealt with. This data answers the first research question and the data from the interviews was used to answer the rest of the research questions.

The data from the interviews were analyzed by using qualitative inductive content analysis. The analysis was done step by step and began with transcribing the interviews. Then the entire data was read through several times to get an overview of the content (Vaismoradi *et al.*, 2013). The material was worked through and the data was simplified to find the essence of the content derived from theoretical background and research questions. Earlier studies of shared governance (Porter-O’Grady and Finnigan, 1984; Porter-O’Grady, 1991; Hess, 2004; Hess, 2011) guided the identification of the emerging structures and processes. The text was then categorized to provide a meaningful description of the content. Categories are tentative and step by step deduced and eventually reduced to main categories, as shown in Table I (Vaismoradi *et al.*, 2013).

### Ethical considerations

The project was approved by the study hospital. Finnish research ethical legislation [Finnish National Board of Research Integrity (TENK), 2009] does not demand ethical permission for this kind of interview study, as it does not involve patients, will cause no harm or security risks and does not include intervention in the physical integrity of subjects.

However, in line with the Declaration of Helsinki, all participants took part voluntarily, received written information and signed written consent before interview (World Medical Association, 2013). The meeting documents where received through the chairs of the councils. Audiotapes, notes and consent forms were anonymously coded and securely locked following interviews.

## Findings

The findings are arranged according to the research questions and the data to the first question is derived from the documents of the council meetings and to the rest of the research questions, the data is derived from the interviews.

### Council structures and processes

The categories describing the structures and processes of the councils were members, reaching decisions, matters discussed and the integration of the functioning of the councils. All together 171 persons participated in the councils during the years of 2010-2014. The chair of the councils is usually a professor or a university researcher. Members act as secretaries, taking turns. New members are recruited via intranet every other year. In the Research Council, members are students in the university or degreed Masters of Science. Other councils admit nurses and others interested.

The councils meet once a month or less in the hospital. Members and outsiders can suggest matters for discussion; also the official policies of the organization direct the agenda. Councils can use experts to inform them of various matters. The decisions are reached through open discussion and smaller committees can be selected to prepare certain matters and report back to the council. Matters are discussed according to the agenda and minutes are written of the meeting.

The personnel of the organization are informed of the work of the councils in the Evidence Based News -bulletin that is published a few times a year. Memos of the meetings are sent to nurse leaders and the members of the councils are expected to share the information in their own units.

The councils organize different types of continuing education for the personnel of the organization. Most of the time spent in the meetings was used to plan and revise these educational opportunities. Issues dealing with the normal functioning of the councils, such as changing the chairperson or secretary, selecting new members, and writing the annual reports took an abundance of time.

We suggest, that the participants from the next EBP training would be made members of the Council because we still need new members to replace the old ones. Document: EBP Council 2011

Scientific articles and ideas to promote evidence based nursing in the organization were discussed. Issues dealing with student guidance were dealt with by The Student Guidance Council.

Evidence based nursing should be made easier approachable and a part of the everyday practice on the wards and the council members should promote this by their own actions. Document: EBP Council 2012

### Factors influencing the functioning of the councils

According to the nurses, there are three factors supporting and four factors obstructing the functioning of the councils (Table I). The strong support of the nurse leaders of the organization was thought to be the main supporting factor of the councils.

The situation here is that we have excellent nurse leaders, they have had the vision, they want to develop things, they see the chance. Interview 4

The organization had recently opened a unit for teaching and developing nursing where clinical experts can give support to the councils. The organization is also offering educational opportunities in evidence-based-nursing and the personnel were seen over all to be enthusiastic about developing nursing care.

[…] members are very motivated and have an interest to the matter. We have a few nurses by the bedside that have a higher degree education and are interested in developing nursing care. They are taking part in the work of the councils. Interview 4

The idea of developing evidence-based-nursing in the organization is evident in the strategy of this Health Care District and it guarantees systematic leadership. The neighboring university has played a large role in contributing to the councils.

The participants felt that lack of time was the main obstructing factor to the councils. Sometimes personnel cannot take part in the meetings during their work hours and do not have time to prepare matters for the meeting. They also felt that their colleagues did not always understand the meaning of the councils and therefore questioned the need to use work hours for the meeting.

[…] they have had to justify the use of work hours and doubted themselves if it is all worth it. Interview 5

As a rule, the idea of evidence-based-nursing was not familiar in the everyday work of the personnel. That led to a lack of interest and will to commit to it.

[…] they don’t seem to understand that it could serve my work. That reflects into not being interested and willing to commit to it, like taking part in the work. Interview 12

Lack of skills needed to find, address and apply information was also seen to obstruct implementing evidence-based-nursing.

### Benefits of councils as part of shared governance

The participants felt that councils benefit the organization in many ways (Table II). They enhance the quality and safety of patient care and facilitate work rotation of the nurses by standardizing nursing practices. They were thought to be best visible in various education opportunities available for the personnel, and nurses felt that the councils offered a way to inform nurse leaders of the problems noticed at the bed side.

[…] if the managers would use the councils to their advance they could direct the discussion into certain themes and get good material to support the decisions making. Interview 9

The councils were either seen as integrating well into the organization or not to integrate at all. Most of the nurses felt that the councils integrated well into the work of the organization. Nursing is a big part of the mission of the organization and the councils were seen to strengthen the visibility of nursing and nursing research, and to bring forth current research questions, as well as spread vital information in the organization. The Councils offer a venue for the personnel to discuss common issues and support the implementation of Best Practices.

I know that it is very tightly integrated into the work of the hospital, because nursing is such a big part of the whole work of the hospital. Interview 6

The councils were seen to improve the image of the organization as an employer by supporting structures that enable personnel to express their thoughts and ideas and further one’s career. It was be seen as making the organization a magnetic place to work. Active development in nursing was also seen to be a pivotal role of a teaching university hospital. Most of the participants felt that councils offer possibilities to have an impact on their work and accountability for developing new ways of thinking. Nurses can show their personal interest in matters and their willingness to develop nursing.

I can make a difference, I can take part, I can be a part of what is happening in our organization. That is a huge benefit. Interview 4

That can then lead to better work opportunities and be personally empowering. Engaging in the councils with others can strengthen the sense of community and therefore ease every day work and enable nurses to see the bigger picture in the organization.

### Developmental needs of councils

Nurses had different kinds of development ideas. The councils should be made more visible in the organization. This could be achieved by better informing personnel about the councils and by bringing evidence-based-nursing into the everyday work. Council members should bring more information from the councils into the wards and research articles should be made available in Finnish in order for them to be easily applied.

[…] every council member should inspire their own ward and tell about the work in different meetings. Document: Research Council 2013

The respondents wanted to see more results from the councils. This could be achieved by optimizing the performance of the councils and by concentrating the discussion into solving everyday problems rather than discussing more general issues. This would call for tightening the role of the nurse leaders to ensure that the councils would concentrate to strategically important matters. More support from the supervisors was also expected to enable personnel to take part in the councils.

The nurse leaders are very important because they make it possible for personnel to take part and as many as possible to take part. Interview 1

The respondents wanted to see development in the working methods of the councils, for instance, they felt that publicizing skills would be needed. They would also want to see different disciplines taking part in the councils, such as therapists, doctors, as well as patients. Furthermore, they hoped that decision-making would be more shared in the organization, and that nursing governance would be one of the issues developed.

## Discussion

This study is one of the first studies of this topic in Finland. The purpose of this study was to describe council structures, its benefits, supportive and obstructive factors and developmental needs as a part of shared governance in one university hospital. We set about this task by reading through meeting documents and interviewing participants.

Councils as a form of shared governance are relatively new in this country and are still seeking form. It has taken courage for the nurse leaders in the study hospital to find a way to make the councils work. The strong support of the nurse leaders has earlier been seen important for functioning of the councils (Brody *et al.*, 2012; Burkman *et al.*, 2012; Meyers and Costanzo, 2015; Lindell Joseph and Bogue, 2018). Participant in the current study were adamant that without nurse leaders clear guidance, the councils would not exist.

Creating a new way to do things takes time. The lack of time was seen to be the main obstructing factor to the functioning of the councils. The participants felt that their colleague nurses did not always understand the meaning of the councils and therefore questioned the need to use work hours for the meetings. The nursing staff’s difficulty to understand the meaning of the councils is a general phenomenon (Palaka *et al.*, 2011; Profit Newman, 2011; Wheeler and Foster, 2013).

Even though the councils in the study hospital are in an early state, many positive results can already be seen. The councils were seen to strengthen the visibility of nursing and nursing research, and to bring forth current research questions, as well as spread vital information in the organization. The results suggest that networking of the personnel supports the functioning of the organization as a whole. Engaging in the councils with others can strengthen the sense of community and therefore ease every day work and enable nurses to see the bigger picture in the organization.

Positive results can also be seen in improvement of the quality of care, harmonization of nursing practices and better informed decision-making. Personnel are having more opportunities to express their opinions and further their expertise and careers, as also seen in earlier litterature (Burkman *et al.*, 2012; Clavelle *et al.*, 2013; Li *et al.*, 2013; Wheeler and Foster, 2013; Yang *et al.*, 2013; Powers and Thornton Bacon, 2016).

The study hospital has its own and unique model of councils and the work processes that work well in that organization. The data from the documents reveals that the work of the councils is arranged hierarchically. A professor or a university researcher act as chair and members are voluntary nursing staff. Maybe next step would be to develop the work processes so that the leadership positions are given to staff nurses as a way to empower them more. Opening the discussion in the councils to all professionals in the care team would also strengthen the work processes. To develop shared governance further in the study hospital, the presence of shared governance in the hospital should be measured. This could be done for example by using The Index of Professional Nursing Governance (Hess, 2017; Weaver *et al.*, 2018).

### Limitations

There are several limitations to this study. First, the sample size was small and second it was not random, but rather based on suggestions from other participants, which may have caused bias by eliminating nurses with more critical opinions about the councils. The study concentrated to describe the work of the councils in one hospital and as such does not claim to be able to replicate or generalize these findings.

Despite the noted limitations, this study adds value to the existing literature, as it describes one way of realizing shared governance in Nordic nursing.

## Conclusions

Our results indicate that the councils as a part of shared governance are promising method to involve nurses to develop nursing. It is also a way to give an option for the nurses to participate in decision-making. Our study shows that the councils produce benefits to the organizational level, nursing and patient care. In addition, councils are important to the development of nurses’ career. Councils are only an example of shared governance and it is import to study other views. In future, the international comparative research about councils is needed.

## Practical implications

The study hospital has its own, unique, council structure. It did not cover the whole hospital or all of the nursing personnel, but it is already producing promising results. It should be given an official status and more support from nurse managers and it should be developed into interprofessional discussion.

The results presented here indicate that shared governance, even, in its early stage, contribute positively to the quality of care, harmonize nursing practices, and informs decision-making. Applying shared governance structures into an organization improves the professional practice environment of nursing personnel. The study showed concrete supporting and obstructing factors that should be notified in nursing leadership.

## Figures and Tables

**Table I.** Factors influencing the functioning of the councils

Sub-category	Category	Theme
Working together		The factors supporting and obstructing the work of the councils
Enthusiastic personnel	Factors supporting the work	
Support from the leaders		
Lack of time		
Lack of understanding		
Lack of skills	Factors obstructing the work	
Resistance of change		

**Table II.** The benefits of councils as part of shared governance

Sub-category	Category	Theme
Unifying of practices		
Networking of the personnel	Benefitting the organization	
Improving of the image		
Support to the decision making		
Improvement of the quality of care		
Making the work of the nurses visible	Benefitting nursing care	The benefits of the work of the councils
A Chance to make an impute		
Improving expertise		
Benefitting ones career	Benefitting personnel	
